# Correcting for the Inflated Adult Population Denominator in an English Nationwide Health Care Cohort: Database Analysis Study

**DOI:** 10.2196/64788

**Published:** 2025-10-27

**Authors:** Sudhir Venkatesan, Mark Joy, Gavin Jamie, Debasish Kar, Robert Williams, Xuejuan Fan, Wilhelmine Meeraus, Ruby S M Tsang, Kathryn S Taylor, Sylvia Taylor, F D Richard Hobbs, Sneha N Anand, Chris Robertson, Simon de Lusignan

**Affiliations:** 1 BPM Evidence Statistics, BioPharmaceuticals Medical, AstraZeneca, Cambridge, United Kingdom; 2 Nuffield Department of Primary Care Health Sciences, University of Oxford, Radcliffe Observatory Quarter, Woodstock Road, Oxford, OX2 6GG, United Kingdom, 44 1865 617855; 3 Faculty of Health, University of Plymouth, Plymouth, United Kingdom; 4 Vaccines and Immune Therapies, BioPharmaceuticals R&D, AstraZeneca, Cambridge, United Kingdom; 5 Population Health Sciences, Bristol Medical School, University of Bristol, Bristol, United Kingdom; 6 Department of Mathematics and Statistics, University of Strathclyde, Glasgow, United Kingdom

**Keywords:** general practitioners, pandemic planning, COVID-19 vaccines, census, selection bias, emergency medical services, national, service, NHS, weighting, record, size, population, registration, register, Health Informatics

## Abstract

**Background:**

Electronic health care databases are widely used for epidemiological studies. However, they may contain inactive records of individuals no longer participating in the health care system. These inactive records create a methodological challenge as they systematically appear as unexposed with no recorded outcomes. Given the widespread health care system engagement during the COVID-19 pandemic, the English National Health Service (NHS), which hosts a national pandemic planning and research dataset with linkage to COVID-19 vaccination and emergency care data, makes it an ideal setting to identify the extent of overrepresentation due to inactive health care records and assess ways to mitigate them.

**Objective:**

The objective of this study is to report any differences between the general practitioner–registered adult population size based on health care records compared to census estimates for England and to apply methodology that could be used to correct for such differences.

**Methods:**

We compared the number of adult patients within the General Practice Extraction Service Data for Pandemic Planning and Research (GDPPR) with a valid general practitioner registration as of 1st October 2021, with estimates published by the Office for National Statistics (ONS) for the English population. We used an approach adapted from a weighting method to correct for non-response bias in surveys and down-weighted individuals with no evidence of recent activity in their records.

**Results:**

There were 61,194,033 registered NHS patients (in the GDPPR) compared with 56,550,138 in the ONS census-based population. De-duplication on NHS number reduced the population to 57,876,641, including 46,835,968 adults, with the biggest overrepresented group aged 30‐45 years. Of the 46,835,986, 1,121,954 (2.4%) individuals had their initial weights down-weighted due to non-engagement with the health care system since January 2019. The down-weighting removed most of the differences between NHS and ONS populations.

**Conclusions:**

There are notable differences in the adult population size as per GDPPR when compared to census estimates. While the overall population size in the GDPPR data was seen to be inflated when compared to ONS census estimates, this was differential with respect to sociodemographic variables. A weighting-based approach can be applied to correct for the inflated denominator. Not correcting for it in large health care datasets, including the English NHS data, could introduce selection bias in epidemiological studies.

## Introduction

Electronic health care databases are widely used resources for conducting epidemiological studies, offering advantages of potentially large sample sizes, reduced costs, and rapid analysis compared to prospective designs. These databases contain longitudinal patient records from routine clinical practice, enabling researchers to evaluate epidemiological associations in real-world settings across diverse populations. However, the secondary use of data collected primarily for administrative or clinical purposes introduces unique methodological challenges that must be addressed to ensure valid inference [1].

An important challenge in database studies is the presence of inactive records—electronic records of individuals who are no longer active participants in the health care system but remain in the database without formal disenrollment. Unlike traditional loss to follow-up in prospective studies where researchers are aware of missing data, inactive records in electronic databases create a particular form of selection bias because they continue to contribute person-time to analyses despite no longer being under observation. While studies have recognized the issue of inactive records potentially inflating the denominator for database [2-4], the precise extent of the issue has not been fully assessed. The COVID-19 pandemic in England offers a valuable opportunity to study denominator inflation, particularly given the high levels of health care engagement observed during this period [5]. England’s National Health Service (NHS) provides comprehensive state-funded health care—including primary, community, and specialist services—to approximately 56 million people, creating an ideal setting for examining this methodological issue.

NHS care includes the provision of medications and the administration of nearly all vaccinations. All NHS patients have a unique personal identifier, an NHS number, which allows records to be linked across NHS services. Additionally, primary care is a registration-based system, with each patient registered with a single general practitioner (GP) [6,7]. Furthermore, NHS computerized medical records (CMRs) have been in use in primary care since the 1980s [8]. Additionally, since 2011, there has been automatic transfer of CMR between practices, even when they use different brands of computer systems [9].

During the COVID-19 pandemic, England was one of the first health systems to use COVID-19 vaccines widely, with high levels of COVID-19 primary series vaccine uptake in adults [10]. The NHS has made a large nationwide cohort available for research through NHS England’s trusted research environment (TRE) [11]. This cohort included the NHS primary care data collections, known as the General Practice Extraction Service Data for Pandemic Planning and Research (GDPPR), which was linked at the individual level to COVID-19 vaccination, COVID-19 virology test results, hospital, and death certificate data.

The discrepancy in population size between GDPPR and Office for National Statistics (ONS) census estimates has been pointed out before [4]. The phenomenon, in NHS data, where an individual may have multiple unique health care records because of not notifying the health care system when they moved to a different GP practice [4,12,13] is known by different terms in the literature, including over-registration, “ghost” patient records, and inactive records. We subsequently refer to such records as ‘inactive records’ in this paper. We aimed to assess the extent of any potential discrepancy in population sizes between the NHS England GDPPR dataset and the ONS census estimates (eg, due to over-registration) and then develop and implement a method to minimize the impact of this discrepancy for epidemiological studies.

## Methods

### Data Source

We used de-identified routinely collected electronic health care record data from primary and secondary care in England accessed via the NHS Digital TRE. Access to the NHS Digital TRE was obtained through a Data Access Request Service (DARS) application. The linked data used for this analysis included GP registration data from GDPPR [10], COVID-19 Second Generation Surveillance System from Pillar 1 and Pillar 2 for COVID-19 PCR testing data, COVID-19 vaccination status data, and Hospital Episode Statistics. Linked and pseudonymized patient-level data were provided to us by NHS Digital within the secure TRE. Details on the data flow, end-to-end linkage process, and data privacy can be found on the NHS Digital website [14]. We used data as of October 1, 2021, for our analyses. In this linked dataset, patient age and sex were obtained from the GDPPR and Personal Demographics Service. The other variables used in this analysis (COVID-19 status, vaccination status, and health care engagement) were defined as yes or no binary flags based on the information from the relevant data source (Second Generation Surveillance System for COVID-19 status, Hospital Episode Statistics for hospital admission).

We assessed the total number of unique, pseudonymized patient identifiers with a valid GP registration as of October 1, 2021, and compared it to estimates published by the ONS for the English population over a comparable time period [15,16]. We refer to this overall GP registration estimate as the “population denominator,” as this will constitute the source population that study-specific cohorts will be constructed from in epidemiological studies that will use these CMR data. We then implemented a method for the denominator correction based on the approach used in a Scottish vaccine pharmacovigilance study [17,18] which is, in turn, based on a weighting method to correct for non-response bias in surveys [19]. We adapted this method to apply to the NHS England data.

#### Step 1

We obtained data from NHS England from GP practices in England as of 1 October 2021. Duplicate records and records missing age and sex were excluded. We used age, sex, and NHS region as the sociodemographic variables for the population weighting. These variables were chosen on the basis that these are some of the key determinants of the differential representation of population groups in the NHS England data [12].

#### Step 2

We restricted these analyses to those aged 18 years and over for consistency with the UK COVID-19 vaccine rollout policy and also due to difficulties with validating children’s GP registration data.

#### Step 3

We obtained population proportions from the ONS on the projected mid-2020 English population stratified for each year of age (≥18 y) for males and females by the following NHS regions: East of England, London, Midlands, North East and Yorkshire, North West, South East, and South West.

#### Step 4

Each individual record in the NHS England analytic dataset was then assigned a weight to match the target distributions obtained from Step 4 such that the weights add up to the target population size (ie, England’s mid-2020 population). These weights were derived using age, sex, and NHS region. For each individual, k, within the NHS England population, the initial study weight, w, was calculated as:


$$w_{ijrk}=\frac{N_{ijrk}}{n_{ijrk}}$$

where N is the number of individuals in the ONS census population in age group i with sex j and region r, and n is the corresponding figure for the NHS Digital population.

#### Step 5

Patient records with recent health care system engagement, defined as *any* of the following since 1st January 2019: GP consultation, hospitalization, laboratory test results, COVID-19 testing, and COVID-19 vaccination were reweighted to 1, as such an encounter would confirm that the individual health care record is active.

#### Step 6

Weights for those patient records without a recent health care system engagement were updated such that the total revised weights (in the overall NHS England data) sum to the ONS estimate of the population of England age ≥18 years. For each individual, k, in age group i with sex j and region r, the revised weight, w*, was calculated as follows:


$$w_{ijrk}=\left(\frac{S_{w}-S_{h}}{S_{w}-S_{w_{nh}}}\right)$$

where S_w_ is the sum of the original weights (and therefore equals the total ONS population size), S_h_ is the sum of the updated weights for individuals with a recent health care engagement (as defined in Step 5), and S_w_nh_ is the sum of the original weights (assigned in Step 4) of individuals without a recent health care engagement.

A validation of the revised weights was performed by comparing the patient profile (in terms of age, sex, and region) of the unvaccinated population in the NHS England data to the unvaccinated patient profile information provided by the ONS [20].

Finally, we also conducted an illustrative bias analysis to demonstrate the impact of the bias introduced by not accounting for inactive records in a hypothetical vaccine effectiveness (VE) study.

### Ethical Considerations

We undertook this work as a part of the RAVEN study, which investigated the effectiveness of the AZD1222 vaccine using CMR from England (Real-world Oxford/AstraZeneca Vaccine Effectiveness Study in England; NCT05047822) [21].

## Results

**Figure 1.**
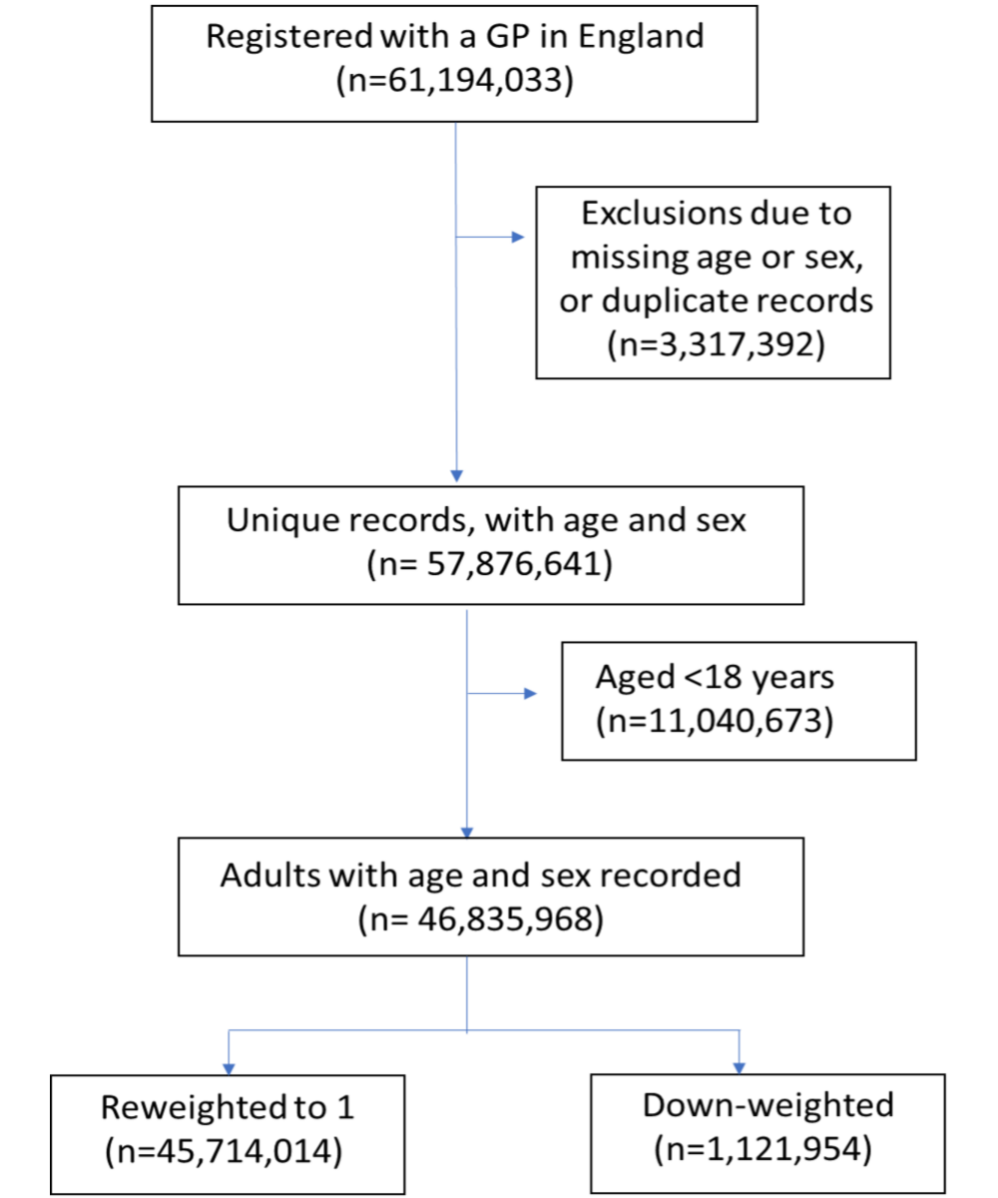
Study flow diagram showing exclusions at each stage.

**Figure 2.**
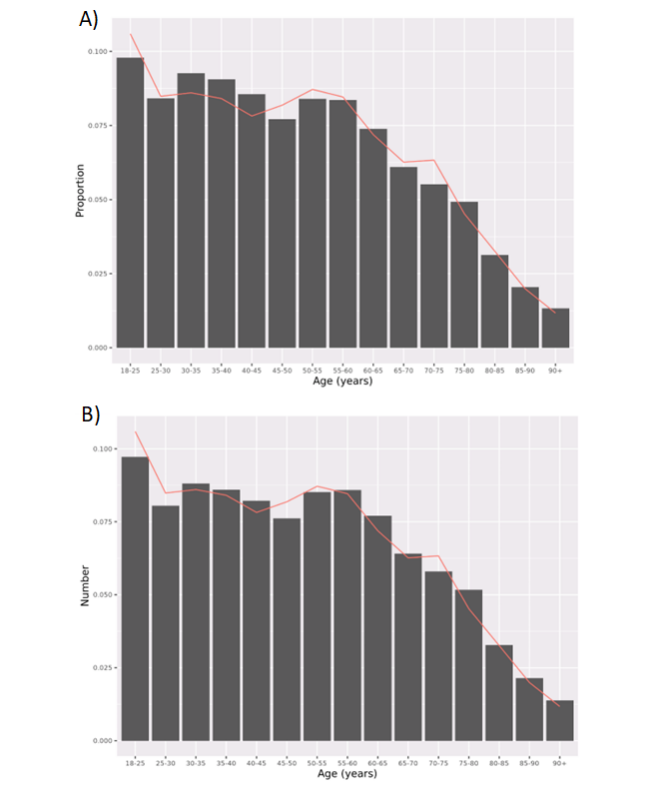
Age distribution (by age group) of the NHS England data (columns) compared to ONS Census data (line) (A) before and (B) after applying weights for inflated denominator correction.

There are 61,194,033 individuals registered at GP practices in England as of 1 October 2021, according to NHS England [22]. However, the ONS estimated the population of England to be 56,550,138 in mid-2020 (including 44,456,850 records for individuals aged ≥18 y) [23]. After de-duplication (ie, removing duplicate records with the same pseudonymized patient identifier) and removing records with missing age or sex, the NHS England data had a total of 57,876,641 unique NHS numbers (including 46,835,968 records for individuals aged ≥18 y) (Figure 1). Age-stratified (categories of age) counts of the NHS England population when compared to ONS estimates revealed that individuals aged 30‐45 years were the most overrepresented groups, and those between 65 and 75 years were among the most underrepresented (Figure 2).

A total of 97.6% of the NHS England adult population had their initial weights revised to 1 on the basis that they had had some form of engagement with the health care system. Of 46,835,968 individuals, 1,121,954 (2.4%) had their initial weights down-weighted due to non-engagement with the health care system since January 2019. The median (IQR) age of the overall NHS population (≥18 years) was 48 years (18-63), whereas the median (IQR) of the down-weighted population was 36 years (18-44). Of the 46,835,968 NHS England records (population aged ≥18 years), 82% had a record of at least 1 COVID-19 vaccination, 62% had a record of a COVID-19 test, 97% had a recorded GP visit, 28% had been admitted to hospital at some point, and 24% had a record of a lab result (any lab test for any condition) since the 1st of January 2019.

Once the final weights were derived, the magnitude of the overrepresentation of the 30‐45 years age category was decreased, although some degree of overrepresentation in these age groups still persisted (Figure 2).

**Figure 3.**
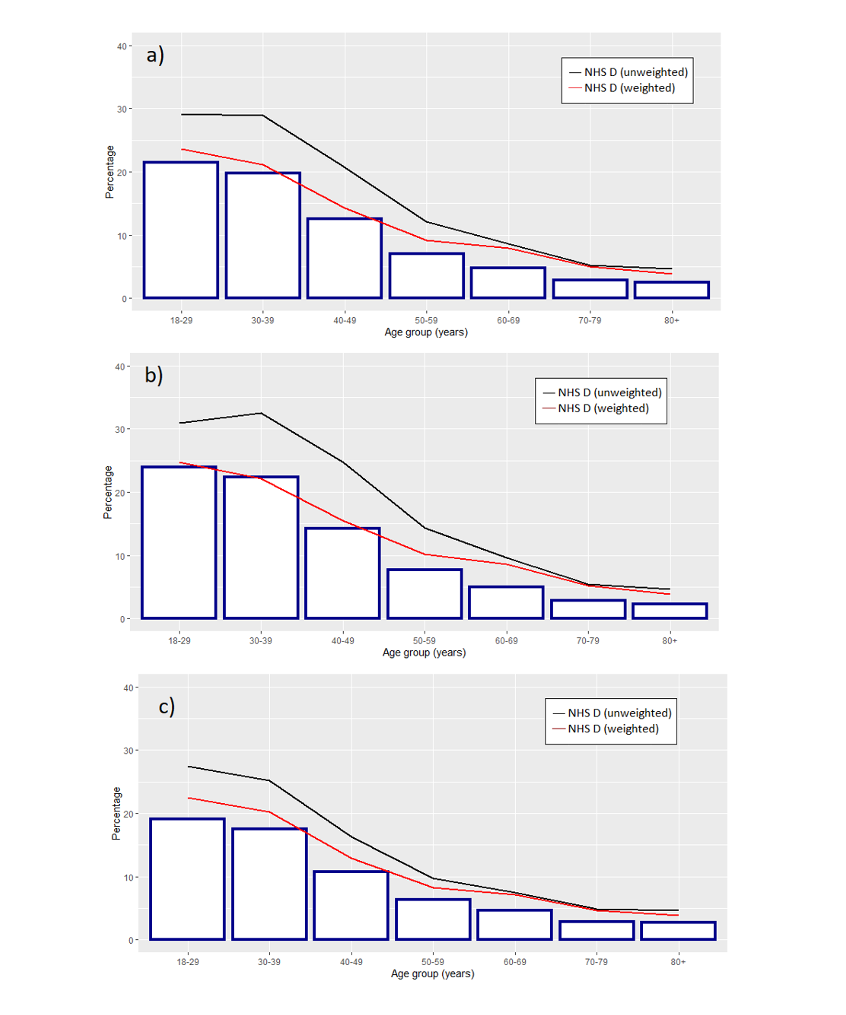
Comparison between the age distributions in the NHS England weighted and unweighted data and the ONS published data among unvaccinated individuals in (A) the overall population, (B) males, and (C) females. NHS D: GDPPR data from the NHS Digital Trusted Research Environment*.*

To assess the impact of the denominator correction, we compared the age and sex distribution of the weighted (denominator corrected) population to the unvaccinated patient profile published by the ONS (in individuals aged ≥18 years between 8/12/2021 and 31/12/2021) based on a subset of the national population [20]. Overall, large differences were seen between the unweighted NHS England data and the ONS estimates. These differences were largely minimized when using the denominator-corrected weighted NHS England data. The most overrepresented group in the NHS England data appeared to be those aged 30‐45 years (Table 1). While most of the overrepresentation in the age groups between 18 and 49 years was seen to have been corrected for when using the weighted data, some degree of overrepresentation was still seen in the age groups between 50 and 80+ years (Table 1, Figure 3, and Multimedia Appendix 1).

**Table 1.** Comparison of age distribution of the NHS England data compared to ONS census data before and after weighting.

Age, years	ONS (%)	NHS Digital	
		Unweighted (%)	Weighted (%)
18-25	10.6	9.8	9.7
25-30	8.5	8.4	8.1
30-35	8.6	9.3	8.8
35-40	8.4	9.1	8.6
40-45	7.8	8.6	8.2
45-50	8.2	7.7	7.6
50-55	8.7	8.4	8.5
55-60	8.5	8.4	8.6
60-65	7.2	7.4	7.7
65-70	6.3	6.1	6.4
70-75	6.3	5.5	5.8
75-80	4.5	4.9	5.2
80-85	3.2	3.1	3.3
85-90	2.0	2.0	2.2
90+	1.2	1.3	1.4

In our bias analysis, for a hypothetical VE study, we assumed an analytic dataset of 10,000 individual records. In Scenario A (reference case), we correctly excluded all inactive records, analyzing only the 9700 active patients. With 60% vaccination coverage, a 1% event rate among vaccinated and a 5% event rate among unvaccinated individuals, we calculated a true VE of 70%. In Scenario B, we incorrectly included 300 inactive records (3% of total) in the analysis, all classified as unvaccinated with no events. This inflated the unvaccinated denominator to 4180 while keeping the event count unchanged, resulting in an underestimated VE of 67.7%, representing an absolute bias of 2.3% (−3.3% relative bias). In Scenario C, we implemented the weighting approach described in this paper, where inactive records received a mean weight of 0.2 and active records a mean weight of 0.9. This approach effectively reduced the contribution of likely inactive records while maintaining most of the influence from active records. The weighted analysis yielded a VE estimate of 69.5%, much closer to the true VE seen in Scenario A with only a 0.5% absolute bias (−0.7% relative bias). A higher level of denominator inflation will be associated with a greater degree of absolute bias in VE estimates if inactive records are not accounted for (Multimedia Appendix 2).

## Discussion

### Main Findings

Our assessment of the NHS England GDPPR data showed that the population size of individuals registered with a GP in England was inflated overall, but specific age groups were underrepresented when compared to the ONS census estimates. The differential nature of the discrepancy could cause certain demographic groups to be over- or under-represented in the NHS England data, which in turn has the potential to influence the results of epidemiological studies that use these data. Possible reasons for “over-registration” and the presence of these inactive records include young, mobile individuals who may have moved out of the country. The NHS number was introduced in 1995; its precise structure and systematic use within NHS information systems were made mandatory in 2009 [24]. Prior to this, multiple registrations were possible. Over-registration has also become much less of a problem since GP2GP transfer of records was introduced in 2011 [9].

### Comparison With Other Studies

Inactive electronic medical records are known to be correlated strongly with sociodemographic factors [12,25], which is a finding that our study corroborates. While methods proposed to adjust for this inflated denominator include data-cleaning exercises [12] and identifying an “active patient” denominator through a validation sample [2], the success of these methods relies on selecting a representative validation sample, and given the large sociodemographic and spatial variation in denominator inflation, this may not be feasible. The COVID-19 pandemic period, which saw nationwide engagement with the NHS, could provide a unique opportunity to infer the “active” status of a patient’s records. We have described a method to first identify the extent of the denominator inflation by inferring “active” registrations through engagement with the health care system (ie, GP consultation, hospitalization, laboratory test result, COVID-19 testing, or COVID-19 vaccination) during the pandemic period and then to correct for it by reducing the weights of those records that did not have any engagement with the health care system during this period. A similar weighting approach that was derived based on population age and sex distribution has been successfully implemented in a series of COVID-19 VE studies based on Scottish data [17,26,27]. We adapted to the English context by also taking regional variability into account. We note that in cohort profiles for OpenSafely and Clinical Practice Research Datalink, similar differences are seen between the adult age-sex profile and ONS data [28,29]. In this paper, we specifically looked at the NHS data in England; however, this issue is a wider problem that could occur in other secondary health care databases as well.

### Implications

Not properly accounting for the inflated denominator in NHS England data could introduce selection bias if it results in follow-up time being differentially accrued in one of the study arms. We illustrated the impact of inactive medical records on a hypothetical VE study in a bias analysis. Inactive records systematically appear as unvaccinated, since they cannot receive documented vaccinations, and without recorded outcomes, since events cannot be documented. This biases the association between vaccination and the outcome of interest by artificially inflating the denominator of unvaccinated individuals without a corresponding increase in events, leading to underestimation of event rates in the unvaccinated group and consequently underestimation of VE. The weighting method that we have proposed seeks to address this bias by decreasing the amount of information contributed to by records with lower weights, which are more likely to be inactive records. Our approach made the observed vaccination and outcome status more closely approximate to the true vaccination and outcome status. The extent of this selection bias will depend on the degree of denominator inflation and also on the sociodemographic factors that the inflation is differential with respect to. The success of the weights in minimizing the impact of selection bias from inactive records will depend on the extent to which they can approximate the true vaccination and outcome status.

We identified an average of 2.4% inflation in the population aged ≥18 years, with this being much higher within specific sociodemographic groups. Indeed, population underrepresentation (as seen in the 65‐75 y age group) would also result in a similar type of selection bias. Our weighting approach accounts for both underrepresentation and overrepresentation using the same method, ie, reducing the weights of those records without a recent health care system engagement during the COVID-19 pandemic.

### Strengths and Limitations

A strength of the weighting approach that we propose is that these individual records are not discarded entirely. The estimated weights can be thought of as confidence scores for each record, and when taken into account in a weighted regression model, they still allow the down-weighted records to contribute information to the model but minimize the impact of any selection bias. The weighting method that we have applied sits within the paradigm of inverse probability weighting methods that are widely used to minimize confounding in epidemiological studies [30]. Current approaches to addressing the impact of inactive records have notable limitations. Complete case analysis that attempts to exclude inactive records often relies on arbitrary rules, such as requiring a minimum number of health care encounters within a specified period. However, such approaches may inadvertently exclude truly healthy individuals with legitimately low health care utilization, introducing another form of selection bias. Alternatively, ignoring the issue by including all records risks substantial underestimation of VE, as our bias analysis demonstrates. Validation samples, where a subset of records undergoes intensive verification through direct patient contact or linkage to external data sources, provide gold-standard identification of inactive records but are resource-intensive and often infeasible at scale. Probabilistic linkage to external data sources (eg, death registries, migration records) can identify specific causes of inactivity but may miss others and introduce complex data governance challenges. Statistical multiple imputation approaches handle uncertainty in inactive status but rely on correctly specified imputation models and may be computationally intensive for large databases. Our proposed weighting method offers several advantages: it retains all available data, acknowledges uncertainty in inactive status classification, can incorporate diverse predictors of inactivity, and is computationally efficient even with millions of records. However, it requires careful validation of the weighting algorithm and may be sensitive to misspecification of the weighting model. The optimal approach likely depends on specific study contexts, with hybrid methods potentially offering the most robust solution for large-scale VE studies. This is an avenue for future research.

Our method for denominator correction has other limitations as well: It assumes that reference population estimates used to derive the weights are based on perfect information. The limitations of ONS census estimates are well-known [12], and it is indeed possible that sociodemographic determinants of census completion are correlated with GP registration. Further, we do not include ethnicity and social deprivation in our analysis, which is a limitation of our weighting scheme. There is evidence to suggest that ethnicity and social deprivation are other key determinants of over-registration [12], but explicitly including these variables in the weighting would require that marginal frequencies be available for the variables in both the GP records database and the census data, which were not available for this analysis. The IMD score, which is the national mapping of deprivation, uses postcodes as of 2019, which means newer postcodes since then do not have a mapping [31]. Further, ethnicity recording is not usually complete either and may need supplemental mapping [32], which would bring with it additional complexities when analyzed. The robustness of a weighting scheme that does not explicitly account for ethnicity or socioeconomic deprivation will hinge on the extent to which the NHS region can be used as a proxy for the 2 variables. Finally, the weighting system does not distinguish between genuine inactive records versus active records of individuals who may have chosen not to engage with the health care system during the pandemic, therefore, potentially also assigning a low weight to these individuals.

Our method does not correct for inherent limitations of the reference population data used. We have also limited the analyses presented in this paper to the adult population (≥18 y), as there are challenges with using census data to validate primary care records of children. Also, we have only explored variables in primary care and have not allowed for diagnoses made in secondary care (eg, cases of chronic liver disease), which may compensate for the gap in primary care diagnosis.

It is also worth noting that while the widespread health care engagement that was observed in England during the COVID-19 pandemic provided a unique opportunity to assess the active status of health care records, a similar population-wide engagement is unlikely in the near future. The ascertainment of the size of the inactive population and application of our weighting method could potentially also be extended to non-pandemic periods. For instance, the weighting algorithm could incorporate routine health care utilization patterns as predictors of active status, including prescription refill consistency for chronic medications, attendance at preventive screenings, completion of age-appropriate vaccinations, and regular check-ups for specific demographic groups (eg, prenatal visits, well-child visits, and geriatric assessments). Validation could follow a three-pronged approach: (1) temporal validation comparing algorithm performance across different calendar periods, (2) external validation through linkage with a subset of verified active or inactive statuses from administrative sources, and (3) sensitivity analyses examining how varying thresholds of predicted inactivity affect VE estimates. Researchers should calibrate weights to their specific health care system’s characteristics, accounting for factors like insurance coverage patterns, referral requirements, and electronic record integration across care settings, which all influence the observable signals of patient activity. This methodological challenge presents a rich opportunity for future research to further develop and validate standardized approaches for addressing bias in epidemiological studies due to the presence of inactive records.

### Conclusions

Denominator inflation in large CMR datasets is a known issue. In this paper, we provide tools for identifying an active population in the NHS England data using a system of sampling weights to minimize the selection bias introduced by denominator inflation. We recommend, where appropriate, that epidemiological analyses of large CMR datasets, including the NHS England data, be implemented as weighted regressions taking into account the likelihood of population over- or under-representation. This is especially necessary for closed data access mechanisms on such data where analyses are often performed in specific defined datasets and an assessment of the impact of mis-specifying the denominator may be difficult, or impossible, to undertake.

## Supplementary material

10.2196/64788 Multimedia Appendix 1 Comparison between the age distributions in the NHS England weighted and unweighted data and the ONS published data among unvaccinated individuals in the overall population and stratified by sex.

10.2196/64788 Multimedia Appendix 2 Methods for bias analysis.

## Abbreviations

CMR: computerized medical record
GDPPR: General Practice Extraction Service Data for Pandemic Planning and Research
GP: general practitioner
NHS: National Health Service
ONS: Office of National Statistics
TRE: Trusted Research Environment
VE: vaccine effectiveness
