# Supplementary material for: Correcting for the Inflated Adult Population Denominator in an English Nationwide Health Care Cohort: Database Analysis Study
Source: JMIR Public Health Surveill. 2025 Oct 27;11:e64788. doi: 10.2196/64788 (PMC12559012; doi:10.2196/64788)
Supplement: Multimedia Appendix 1 [file publichealth-v11-e64788-s001.docx]

# Multimedia Appendix 2

Comparison between the age distributions in the NHS England weighted and unweighted data and the ONS published data amongst unvaccinated individuals in the overall population and stratified by sex

|  | **Overall (%)** | | | **Males (%)** | | | **Females (%)** | | |
| --- | --- | --- | --- | --- | --- | --- | --- | --- | --- |
|  | **ONS** | **NHS Digital (unweighted)** | **NHS Digital (weighted)** | **ONS** | **NHS Digital (unweighted)** | **NHS Digital (weighted)** | **ONS** | **NHS Digital (unweighted)** | **NHS Digital (weighted)** |
| 18 to 29 | 21.5 | 29.0 | 23.5 | 24.0 | 30.9 | 24.6 | 19.2 | 27.4 | 22.4 |
| 30 to 39 | 19.8 | 29.0 | 21.2 | 22.4 | 32.4 | 22.1 | 17.6 | 25.1 | 20.3 |
| 40 to 49 | 12.6 | 20.6 | 14.2 | 14.3 | 24.7 | 15.4 | 10.9 | 16.3 | 12.9 |
| 50 to 59 | 7.1 | 12.1 | 9.1 | 7.8 | 14.2 | 10.1 | 6.4 | 9.7 | 8.2 |
| 60 to 69 | 4.8 | 8.6 | 7.8 | 5.0 | 9.6 | 8.5 | 4.7 | 7.5 | 7.1 |
| 70 to 79 | 2.9 | 5.1 | 4.8 | 2.9 | 5.3 | 5.0 | 2.9 | 4.9 | 4.5 |
| 80+ | 2.5 | 4.7 | 3.8 | 2.3 | 4.7 | 3.9 | 2.8 | 4.7 | 4.0 |
