# Supplementary material for: Correcting for the Inflated Adult Population Denominator in an English Nationwide Health Care Cohort: Database Analysis Study
Source: JMIR Public Health Surveill. 2025 Oct 27;11:e64788. doi: 10.2196/64788 (PMC12559012; doi:10.2196/64788)
Supplement: Multimedia Appendix 2 [file publichealth-v11-e64788-s002.docx]

# Multimedia Appendix 1

**Methods for Bias Analysis**

We conducted a bias analysis to quantify the impact of inactive records on vaccine effectiveness (VE) estimates under different scenarios. This analysis examined how the presence of inactive records in electronic healthcare databases affects VE estimates and evaluated the performance of a weighting approach for bias mitigation.

**Assumptions**

For all analyses, we maintained the following core assumptions:

- Total study population: 10,000 individuals
- True vaccination rate among active records: 60%
- True vaccine effectiveness: 70%
- Event rate among unvaccinated individuals: 5%
- Event rate among vaccinated individuals: 1.5% (derived to achieve 70% VE)
- Inactive records appear as unvaccinated with no recorded events

We varied the proportion of inactive records (3%, 10%, and 20%) to assess how the magnitude of bias changes with different levels of record inactivity.

**Statistical Approach**

For each inactive record proportion, we calculated vaccine effectiveness under three scenarios:

**Scenario A: Correct exclusion of inactive records (reference standard)** VE is calculated after properly excluding all inactive records:

VE_A = 1 - (Risk in vaccinated)/(Risk in unvaccinated)

**Scenario B: Incorrect inclusion of inactive records** VE is calculated with inactive records incorrectly included as unvaccinated individuals with no events:

VE_B = 1 - (Risk in vaccinated)/(Risk in unvaccinated + inactive)

**Scenario C: Weighted analysis** VE is calculated using a weighting approach where:

- Inactive records receive a mean weight of 0.2
- Active records receive a mean weight of 0.9
- Each record contributes proportionally to its weight

VE_C = 1 - (Weighted risk in vaccinated)/(Weighted risk in unvaccinated)

**Results**

**Summary Table**

| **Inactive Records** | **Scenario** | **VE Estimate** | **Absolute Bias** | **Relative Bias** | **Bias Reduction** |
| --- | --- | --- | --- | --- | --- |
| 3% | A | 70.00% | Reference | Reference | - |
| 3% | B | 67.67% | -2.33% | -3.33% | - |
| 3% | C | 69.51% | -0.49% | -0.70% | 79.0% |
| 10% | A | 70.00% | Reference | Reference | - |
| 10% | B | 61.64% | -8.36% | -11.94% | - |
| 10% | C | 68.15% | -1.85% | -2.64% | 77.9% |
| 20% | A | 70.00% | Reference | Reference | - |
| 20% | B | 51.26% | -18.74% | -26.77% | - |
| 20% | C | 65.83% | -4.17% | -5.96% | 77.7% |

**Conclusion**

This bias analysis demonstrates that inactive records can substantially bias vaccine effectiveness estimates downward, with the magnitude of bias directly proportional to the prevalence of inactive records in the database. The weighting approach consistently mitigates approximately 78-79% of this bias across different inactive record prevalence scenarios, providing a robust method for addressing this methodological challenge in CHR database studies.
